# Primary Solitary Tuberculosis of the Liver

**DOI:** 10.1155/1992/95242

**Published:** 1992

**Authors:** Ali Emre, Edip Akpinar, Koray Acarli, Aydin Alper, Uǧur Çevikbaş, Orhan Arioǧul

**Affiliations:** 1 Departments of Hepatopancreatobiliary Surgery and Pathology University of Istanbul Medical Faculty Çapa Istanbul Turkey; 2 9-10 Mah. A7B-95 34750 Ataköy Istanbul Turkey

## Abstract

Primary solitary tuberculous involvement of the liver is a rare condition. We present the case of a patient who was operated on with a preoperative diagnosis of hepatocellular carcinoma. Liver resection was performed and antituberculous therapy was started. It is difficult to make the correct diagnosis preoperatively except when a successful needle biopsy can be performed. Despite the rarity of the condition primary solitary tuberculosis should be considered among the space occupying lesions of the liver.


*HPB Surgery*, 1992, Vol. 5, pp. 261–265
Reprints available directly from the publisher
Photocopying permitted by license only

© 1992 Harwood Academic Publishers GmbH
Printed in the United Kingdom

### CASE REPORT

### PRIMARY SOLITARY TUBERCULOSIS OF THE LIVER

ALI EMRE, EDIP AKPINAR, KORAY ACARLI, AYDIN ALPER, UĞUR ÇEVIKBAŞ, ORHAN ARIOĞUL.

*Departments of Hepatopancreatobiliary Surgery and Pathology, University of Istanbul, Medical Faculty, Çapa, Istanbul, Turkey*

*(Received 25 September 1991)*

Primary solitary tuberculous involvement of the liver is a rare condition. We present the case of a patient who was operated on with a preoperative diagnosis of hepatocellular carcinoma. Liver resection was performed and antituberculous therapy was started. It is difficult to make the correct diagnosis preoperatively except when a successful needle biopsy can be performed. Despite the rarity of the condition primary solitary tuberculosis should be considered among the space occupying lesions of the liver.

KEY WORDS: Liver tuberculosis, solitary liver lesions

### INTRODUCTION

Primary solitary tuberculosis of the liver is a rare entity which appears as a space occupying lesion in the liver without a primary focus in another part of the body. Approximately 100 cases have been reported in the literature[1]. Here we report on a young man affected by primary solitary tuberculosis of liver.

### CASE REPORT

A 24 year-old male was admitted to the Hospital complaining of moderate fever and pain in the right hypochondrium. These complaints had started 10 months before and gradually increased. A needle biopsy had been attempted in another hospital but failed. On physical examination, 1 cm hepatomegaly and tenderness in the right hypochondrium were noted. Laboratory tests were all normal except a WBC of $15600/mm^3$ and erythrocyte sedimentation rate of 32 mm/h. Serum alpha fetoprotein and carcinoembryogenic antigen were within normal limits.

No pathological changes were observed in chest X-ray. Ultrasonography revealed a lesion 8 cm in diameter in liver segments VI and VII. Computerized axial tomography showed the same lesion with inhomogeneous contrast enhancement in the right liver. Some areas in the lesion with low density were thought to be

Address correspondence to: Dr A. Emre, 9-10 Mah. A7B-95 34750 Ataköy, Istanbul, Turkey necrotic regions and hepatocellular carcinoma was strongly suspected. No additional pathology was recorded in other abdominal organs.

He was operated on with the diagnosis of a liver tumor. On exploration, a mass, hard and firm in consistency was encountered in liver segments VI and VII. Other organs were all normal and there were no enlarged lymph nodes in the abdomen. A frozen section examination disclosed a benign lesion which was thought to be tuberculosis. Segments six and seven were resected. Histological examination confirmed the diagnosis of tuberculosis (Figures 1 and 2). Antituberculous drug therapy consisting of streptomycin, isoniazide, ethambutol and para-aminosalicylic acid was started and he was discharged after an uneventful postoperative period. No abnormal findings were found by ultrasonography and CAT of the liver and abdominal cavity one month after the operation.

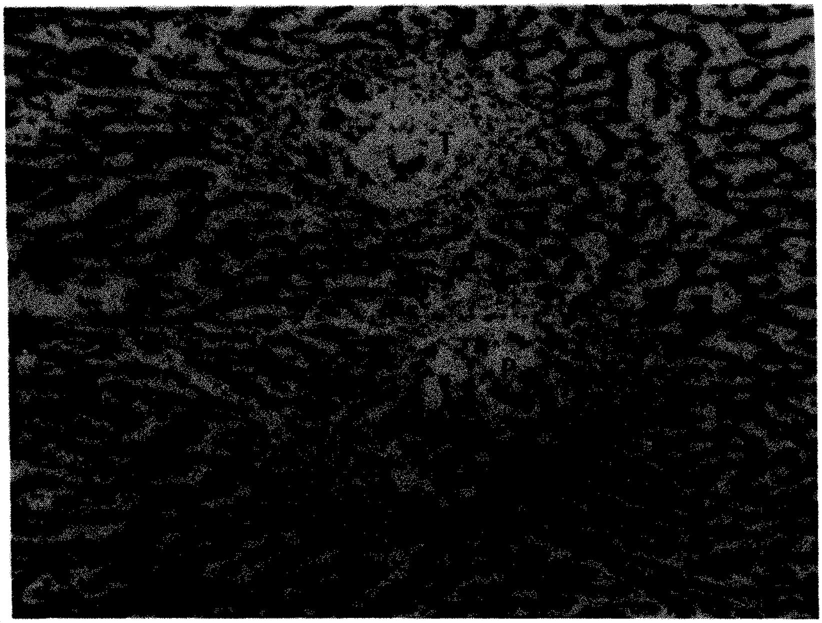

**Figure 1** In paraffin sections tuberculous granuloma (T) and portal area (P) are seen (H.E.X125).

### DISCUSSION

Tuberculosis of the liver generally occurs secondary to tuberculous involvement of other organs such as lungs, intestine etc. and is seen in 3 forms:

1. Diffuse liver involvement concomitent with pulmonary tuberculosis. This is the most common form and encountered in approximately 50-80% of cases who die of lung tuberculosis.
2. Diffuse disease of the liver in the abscence of pulmonary tuberculosis. (Primary miliary tuberculosis of the liver).
3. Tuberculoma and tuberculous abscess. These forms occur as space occupying lesions in the liver and are the rarest form of the disease. They are usually confused with liver abscesses and tumors[1]. In 1965 Gracy[2] found 89 cases in the literature. It has been suggested in recent reports that the total number of cases of primary solitary tuberculosis of the liver does not exceed 100 patients[1]. Actually, the real figure must have been less than this number, because some of these cases also had extrahepatic involvement. Stevens *et al.*[1] reported that they could find only 12 cases in the English language literature. According to Nagai *et al.*[3] only 14 cases could be found in the Japanese Literature. Therefore a precise incidence is not known and more extensive screening is necessary especially in countries where the disease is endemic. Tuberculosis is endemic in Turkey. According to data from the Turkish Ministry of Health, the incidence of tuberculosis in Turkey is 60.5 per 100 000 population[4]. It would seem that this "old" disease is once more becoming a problem, not only in underdeveloped countries, but also in some parts of the developed Western world[5]. It might be postulated that the incidence of liver tuberculosis may increase in the future.

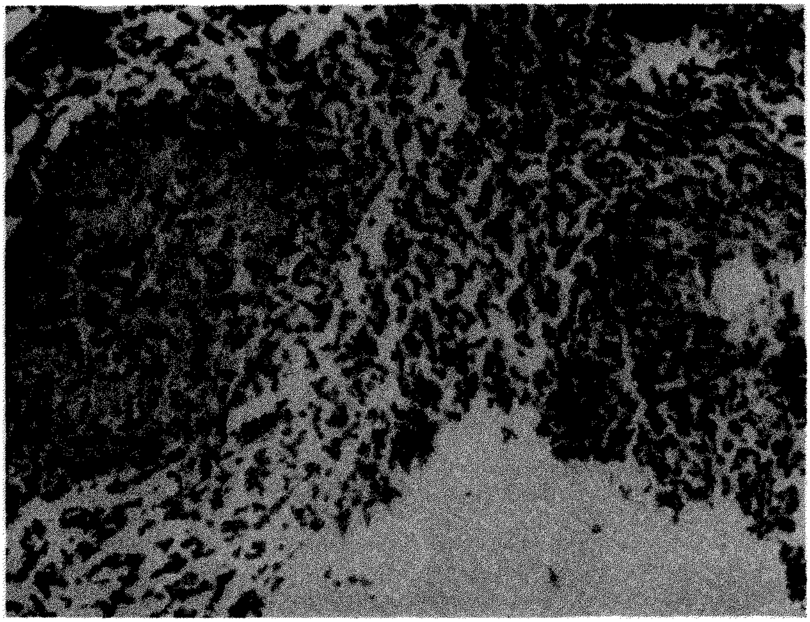

**Figure 2** Two tuberculous granulomas in paraffin sections of the rest liver tissue prepared for frozen section (H.E.X310)

Patients generally complain of fever, abdominal pain, fatigue and weakness. High fever and jaundice may occasionally be present[6,7]. Physical examination may be completely normal. In some cases, hepatomegaly is the only physical finding. Liver function tests are usually within normal limits, although serum alkaline phosphatase levels have been reported to be high in most cases. In our patient it was normal. There are no physical or biochemical findings which are specific or characteristic for primary solitary tuberculosis of the liver.

Both ultrasonography and computerized tomography can display the lesion in the liver but it is generally not possible to distinguish this lesion from other space occupying lesions. According to Nagai *et al.*[3] a hypervascular phase reflecting the inflammatory neovascularization of tuberculoma can be observed on a selective hepatic artery angiogram. Imaging studies in our case have displayed a tumor mass consisting of necrotic areas, with inhomogeneous echoes and contrast enhancement.

A preoperative diagnosis can only be accomplished by successful needle biopsy of the lesion under the guidance of ultrasonography or computerized tomography[1,8,9]. Failure of the first attempt to biopsy the lesion in another hospital, the malign tumor appearance on ultrasonography and CAT and very low probability of primary solitary tuberculosis of the liver in this patient led us to avoid a second biopsy. Even a successful biopsy may not result in a confident diagnosis and most of the cases can only be diagnosed at laparotomy or postmortem examination[1,2,8,10]. In our case, we resected the involved segments after a frozen section diagnosis. It may be disputed whether the operation is finished after a tissue diagnosis and antituberculous therapy is started or whether a liver resection and drug therapy in the postoperative period should be performed. We preferred the latter course. In our opinion, if a definitive diagnosis can be achieved by percutaneous needle biopsy, medical therapy is preferred, otherwise a resection procedure should be performed. After a resection plus drug therapy results are satisfactory, whereas with drug treatment only, successful case reports are very limited.

Postoperative results are excellent. We started antituberculous therapy with a combination of four drugs and plan to continue the treatment for one year. Six months after the operation clinical and diagnostic studies were all normal.

In conclusion, this rare type of tuberculosis should be considered in the differential diagnosis of solitary liver lesions. The ideal treatment is drug therapy if the disease can be diagnosed by needle biopsy. Otherwise surgical treatment should be instituted at least so as not to overlook a malignant lesion.

### *References*

1. Stevens, A. and Little, J.M. (1987) Isolated tuberculous hepatic abscess. *Aus. N.Z.J. Surg.*, **57**, 409–411
2. Gracy, L. (1965) Tuberculous abscess of the liver. *Brit.J.Surg.*, **52**, 442–443
3. Nagai, H., Shimizu, S., Kawamoto, H. *et al.* (1989) A case of solitary tuberculosis of the liver. *Jpn.J.Med.*, **28**, 251–255
4. Yalçin, I. (1989) Prevention of tuberculosis. *Klimik Derg.*, **2**, 25–26 (In Turkish)
5. Reider, L.H., Cauthen, G.M., Kelly, G.D. *et al.* (1989) Tuberculosis in the USA. *JAMA*, **262**, 385–389
6. Abascal, J., Martin, F., Abrev, L. *et al.* (1988) Atypical hepatic tuberculosis presenting as obstructive jaundice. *Am.J.Gastroenterol.*, **83**, 1183–1186
7. Alvarez, S.Z. and Carpio, R. (1983) Hepatobiliary tuberculosis. *Dig.Dis.Sci.*, **28**, 193–200
8. Desmidt, P., Apfelbaum, M., Holroet, J. *et al.* (1988) Tuberculous perihepatic abscess, *Acta Clinica Belgica*, **43**, 378–380
9. Blangy, S., Cornud, F., Sibert, A. *et al.* (1988) Hepatitis tuberculosis presenting as tumoral disease on ultrasonography, *Gastrointest.Radiol.*, **13**, 52–54
10. Dekhne, R., Moore, W.H., Long, S.E. *et al.* (1987) Tuberculous pseudotumor of the liver, *Clin.Nucl.Med.*, **12**, 816--819

(Accepted by S. Bengmark 24 October 1991)

### INVITED COMMENTARY

Solitary tuberculoma of the liver is a rare entity, and no-one could be expected to gain a large experience of it. We would basically agree with the suggestions made by Dr Ali Emre and colleagues[1]. Diagnosis can be very difficult, and many of the cases of reported tuberculoma of the liver have come from areas where hepatitis B and hepatocellular carcinoma are also endemic. Alpha feto protein is more likely to be raised in hepatocellular carcinoma associated with hepatitis B, but a negative alpha feto protein would not exclude HCC. In the absence of tuberculosis elsewhere, tuberculous abscess is not likely to be high on the differential diagnostic list[2]. We would certainly agree that medical therapy should be tried if the diagnosis is made by biopsy or fine needle aspiration, although there is little information in the literature on the success of this policy. There seems to be no alternative to resection in those who are otherwise fit for surgery if a resectable hepatic lesion is found without the possibility of a definite diagnosis.

### *References*

1. Stevens, A., Little, J.M. (1987) Isolated tuberculous hepatic abscess. *Aust. N.Z. J.Surg.*, **57**, 409
2. Leader, S.A. (1952) Tuberculosis of the liver and gallbladder with abscess formation: a review and case report. *Ann.Int.Mem.*, **37**, 594

J.M. Little
Department of Surgery, Westmead Hospital
Westmead, N.S.W. 2145 Australia

